# Nanophase REE phosphate crystallization induced by vivianite oxidation: mechanistic insights and mineralogical implications†

**DOI:** 10.1039/d4ra08110b

**Published:** 2025-04-09

**Authors:** M. Maddin, L. Terribili, R. Rateau, A. M. Szucs, J. D. Rodriguez-Blanco

**Affiliations:** a Department of Geology, School of Natural Sciences, Trinity College Dublin Dublin 2 Ireland maddinm@tcd.ie; b iCRAG, Department of Geology, School of Natural Sciences, Trinity College Dublin Dublin 2 Ireland

## Abstract

Our study investigates the interaction between multi-component rare earth element (REE; La, Ce, Pr, Nd, Dy)-bearing aqueous solutions and vivianite (Fe_3_^2+^(PO_4_)_2_·8H_2_O) grains under hydrothermal conditions (50–165 °C). The results revealed the solution-mediated, progressive oxidation and dissolution of vivianite. This resulted in the formation of iron phosphates, metavivianite [Fe^2+^Fe_2_^3+^(PO_4_)_2_(OH)_2_·6H_2_O], and giniite [Fe^2+^Fe_4_^3+^(PO_4_)_4_ (OH)_2_·2H_2_O], iron oxide hematite [Fe_2_O_3_], and rare earth phosphates, rhabdophane [REE(PO_4_)·H_2_O] and monazite [(LREE)PO_4_]. The extent of the reactions was found to be dependent on temperature, pH, and the concentration and ionic radii of the rare earths in solution. The rate of vivianite oxidation and dissolution increased with increased temperature, with 50% of vivianite transformed after 32 days at 50 °C, and 100% transformed after 28 days and 4 hours at 90 and 165 °C respectively. The pH of the solutions at all three temperatures maintained the stability of rhabdophane, and only at the highest temperature of 165 °C it began to transform to monazite. Understanding the stability of iron phosphates, their transformation products, and their capacity to incorporate REEs is crucial for resource recovery, especially in the extraction of REEs from waste materials.

## Abstract 2

Our study investigates the interaction between multi-component rare earth element (REE; La, Ce, Pr, Nd, Dy)-bearing aqueous solutions and vivianite (Fe_3_^2+^(PO_4_)_2_·8H_2_O) grains under hydrothermal conditions (50–165 °C).

## Graphical abstract

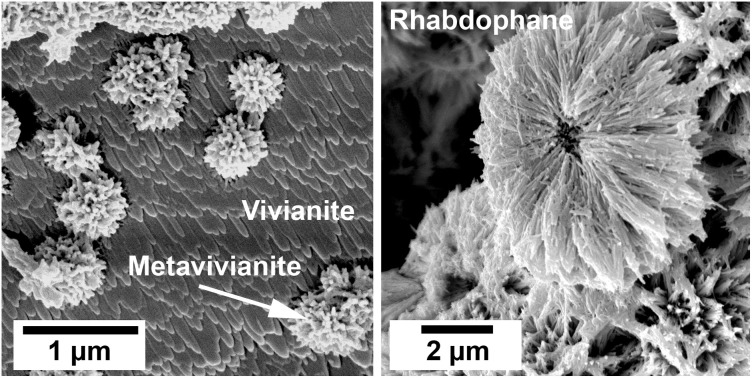

## Introduction

Phosphate rocks are considered to be an essential resource as they provide the raw material for phosphoric acid and fertilizers utilized in agriculture, thereby contributing to the sustainability of the world's food supply. They are also gaining increased interest as a potential rare earth element (REE) source.^1^ With their unique optical, magnetic, and catalytic properties, REEs are becoming more and more essential in modern technologies and are considered critical metals.^2,3^ However, as minable concentrations of REEs are limited, there is a substantial risk to their supply and availability.^4^ REE deposits that contain iron phosphates are of significant interest due to their potential economic value and strategic importance. One example is the Mount Weld carbonatite deposit in western Australia, which has been identified as a secondary rare earth phosphate deposit where phosphates, such as monazite, are enclosed within iron oxide minerals.^5,6^ The deposit, which is enclosed within a weathering cap of an intrusive plug^7^ and contains an average REE grade of 9.8 wt% ∑REE_2_O_3_ and REE concentrations up to 45 wt% ∑REE_2_O_3,_ is now considered one of the richest deposits in the world.^8^ Phosphorite-type rare earth deposits, such as the early Cambrian Zhijin phosphorite, China, are also known for their high concentrations of REEs including yttrium (REEY),^9^ as well as heavy REE enrichment.^10^ During its formation, which was initiated by the breakup of supercontinent Rodinia, the local reducing environment promoted the release of REEY from hydroxides which dramatically increased the concentration of REEY in the seawater. As the supercontinent continued to break apart, there was an upwelling of hydrothermal fluids which carried phosphorous-rich materials. The upwelling processes contributed to the reducing environment and concomitantly to the dissolution of REEs. The upwelling currents carried the P-and REE-rich complexes to the upper oxidising surface waters, where they were able to mix and form REEY-rich apatite.^9^ Studies have also shown a correlation between REE distribution and phosphorous in sedimentary phosphate deposits,^11^ with particular interest in the microdistribution of REEs in specific mineral phases and their locations. Further analysis of the Zhijin deposit by Xiong *et al.* (2024) revealed that the REE distribution was highly correlated with that of phosphorous and that they occur as lattice defects in apatite *via* isomorphic substitution. Lastly, an association of Fe with P and REEs has been observed in high-P ooidal iron ores, with some of the largest resources located in Morocco.^1^ Current research indicates that their formation is linked to large igneous provinces, upwelling, marine hypoxia, and rifting.^12,13^ The recent discovery by the state-owned Swedish mining company LKAB of a significant REE deposit in the Kiruna iron ore mine in north Sweden has the potential to provide a substantial REE source for Europe. LKAB has reported mineral resources of rare earth metals exceeding one million tonnes of oxides, making it the largest known deposit of its kind in Europe. The rare earths at Kiruna occur together with phosphorous in apatite in an iron ore deposit.

In order to better understand the relationship and mineralization pathways between iron phosphates and REEs we chose to react the mineral vivianite (Fe_3_(PO_4_)_2_·8H_2_O) with multi component REE-bearing aqueous solutions at hydrothermal conditions. Vivianite is a common Fe(ii)-phosphate mineral found globally in marine, freshwater, and terrestrial systems.^14^ Its formation in natural deposits is a complex process influenced by redox conditions, sedimentary deposits, porewater interactions, and the presence of key elements such as Fe, P and S. The aim of this study is to investigate whether the interaction of vivianite with rare earth-bearing aqueous solutions would lead to the various processes and/or transformations such as dissolution–recrystallization and oxidation. With REEs present in the aqueous solution, their interaction with vivianite has the potential to result in the formation of rare earth phosphate compounds or solid-solutions. These reactions could involve the precipitation of rare earth phosphates or the incorporation of REEs into the crystal structure of vivianite. It is well known that vivianite has the capability to uptake elements from aqueous solution.^15^ Therefore, the interaction between vivianite and REEs in aqueous solution could potentially lead to the adsorption or complexation of the REEs onto the vivianite surface.^3^ The formation of such compounds could impact the speciation and mobility of the REEs in the aqueous solution. Further research into the specific mechanisms and outcome of these interactions could provide valuable insights into the behaviour of REEs and iron phosphates in natural aquatic and hydrothermal environments. The replacement of iron phosphates by REE-phosphates like rhabdophane, monazite or xenotime would have important implications for resource recovery, particularly in the context of REE from waste materials. Transforming iron phosphates into stable rare earth phosphates can significantly enhance the recovery of economically valuable REEs from sources such as industrial waste and contaminated soils, supporting their critical role in high-tech and green energy industries.

## Methods

The interaction of vivianite (Fe_3_(PO_4_)_2_·8H_2_O) grains with multi-component REE (La, Ce, Pr, Nd, Dy) aqueous solutions was investigated at hydrothermal conditions (50–165 °C). Vivianite crystals were crushed in a ceramic mortar and sieved to extract 0.5–1.0 mm clasts. Five single REE bearing solutions were prepared, La, Ce, Pr, Nd and Dy, with a total concentration of 50 mM. These solutions were produced *via* the dissolution of single REE nitrate salts, REE(NO_3_)_3_·6H_2_O (Sigma-Aldrich, 99.9% purity), in de-ionized water (18.18 MΩ cm). These five specific REE were chosen as they are representative of both light (La, Ce, Pr, and Nd) and heavy (Dy) REE. This group of REE from La to Dy also represent 72% of the ionic radii of the lanthanides as well as being some of the most abundant REE in the Earth's crust.^16^ To better understand the effect of ionic radii of the five REEs and to what extent these ions are taken up by the vivianite host, two different sets of experiments were conducted, one with equal concentrations of the five REEs and one with the concentrations of the five REEs normalized to the commonly used Post Archean Australian Shale standard (PAAS),^17^ to mimic the REE concentrations found in continental crust and in natural geological fluids. For the equal concentration experiments, 4 mL of each of the five REEs were used to give a total solution volume of 20 mL for each experiment. For the PAAS experiments, a 1 L bulk solution was made and 20 mL of this was used for each experiment.

For each experiment, 0.1 g of vivianite grains were added to 20 mL of each of the 50 mM REE-bearing solutions and placed in 25 mL Teflon-lined and capped stainless-steel autoclaves. The reactors were then placed in a pre-heated oven at 50, 90, and 165 °C. Solid samples were then extracted using a sterilized metal spatula at increasing time intervals from 4.5 hours to 7 weeks. The samples were then placed in plastic Eppendorf tubes and dried in a 30 °C oven for at least one hour.

In order to identify and quantify the formation of crystalline solids present in our samples, several grains were selected from each experiment ensuring that each time and temperature variable was represented. The selected grains were ground to a consistently fine powder using an agate pestle and mortar. The minerals were identified and quantified with powder X-ray diffraction (XRD). Conventional powder XRD patterns were collected using a Siemens/Bruker D5000 powder X-ray diffractometer (Cu Kα radiation, 0.01° per step from 5 to 60° 2*θ* at 0.2° min^−1^; 4.5 hours scans per sample). Identification of crystalline phases was carried out with the DiffracSuite EVA software from Bruker in combination with the Powder Data File (PDF-4, the International Centre for Diffraction Data).^18^ Pattern-matching refinement and quantification of crystalline phases were carried out with the Rietveld refinement software TOPAS.^19^ Finally, scanning electron microscopy (SEM) was used to obtain high resolution images to characterize changes in the morphology of the vivianite host grains and identify newly formed phases. Analyses were conducted in the iCRAG Lab at Trinity College Dublin using a TIGER S8000 FEG-SEM operating under high vacuum conditions and equipped with two Oxford X-Max 170 mm^2^ EDS detectors and an X4 pulse processor running the Oxford Aztec analysis software.

Saturation indexes of solid phases during the equilibration of the REE-bearing aqueous solutions with respect to vivianite were calculated with the hydrogeochemical code PHREEQC^20^ using the LLNL database and the solubility products of REE-bearing phosphates and Fe oxides determined by.^21^

## Results

Analysis of all solid samples obtained from the replacement reactions showed the formation of surface precipitates that either partially or totally replaced the vivianite host grain. The combination of powder X-ray diffraction (XRD), scanning electron microscopy (SEM) and energy dispersive X-ray spectroscopy (EDS) allowed us to identify and quantify the newly formed phases and interpret the mechanisms responsible for the alteration and decomposition of vivianite and the precipitation of the newly formed phases.

### Powder X-ray diffraction (XRD)

**Fig. 1:**
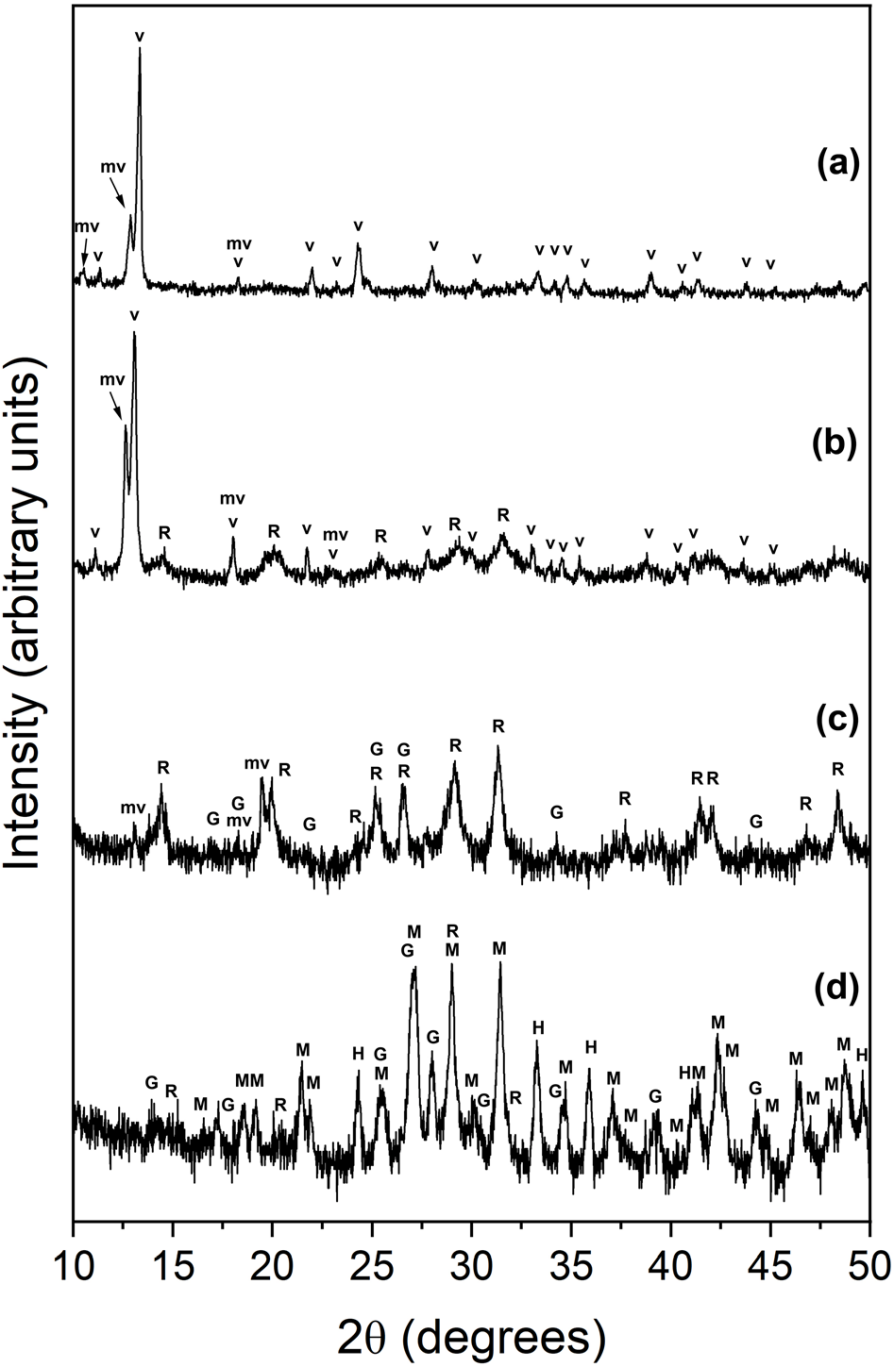
Powder XRD diagrams from samples obtained at (a) 50 °C equal concentration experiments, 24 hours; (b) 50 °C, equal concentration experiments, 32 days; (c) 90 °C, PAAS experiments, 28 days; (d) 165 °C PAAS experiments, 1 week. Bragg peaks have been assigned to the following phases: vivianite (v), metavivianite (mv), rhabdophane (R), giniite (G), monazite (M), hematite (H).

The interaction between vivianite and multi-component rare earth element (La, Ce, Pr, Nd and Dy)-bearing aqueous solutions resulted in the formation of iron phosphates, metavivianite (Fe^2+^Fe_2_^3+^(PO_4_)_2_(OH)_2_·6H_2_O; PDF 00-030-062) and giniite (Fe^2+^Fe_4_^3+^(PO_4_)_4_(OH)_2_·2H_2_O; PDF 00-033-0669), iron oxide, hematite (Fe_2_O_3_; PDF 00-013-0534), and rare earth phosphates, rhabdophane (REE(PO_4_)·H_2_O; PDF 00-035-0614) and monazite ((LREE)PO_4_; PDF 00-029-0403) (Fig. 1). Full quantitative data is available in Table 1.

**Table 1:** Experimental conditions, identities and morphologies of the solid iron phosphates, rare earth phosphates and iron oxides formed during the interaction of vivianite with multi-component (La, Ce, Pr, Nd, and Dy) REE-bearing aqueous solutions (equal concentration and PAAS experiments) at 50, 90, and 165 °C^a^

Equal concentration					PAAS			
*T* (°C)	Time (days)	% phase consumed	Phase formed	Morphology	Time (days)	% phase consumed	Phase formed	Morphology
50	1	28	Metavivianite	Bladed flaky crystals	1	12	Metavivianite	Bladed flaky crystals
	3	30	Metavivianite		3	21	Metavivianite	
	9	16	9% metaviv., 7% rhab.	Metaviv: bladed flaky crystals rhab: flakes & needle-like	9	44	32 metaviv., 12 rhab.	Metaviv: bladed crystals, rhab.: flakes & needle-like
	18	47	13% metaviv., 34% rhab.		18	21	<1% metaviv., 21% rhab.	
	25	57	31% metaviv., 27% rhab.		25	33	<1% metaviv., 33% rhab.	
	32	60	24% metaviv., 36% rhab.		32	45	3% metaviv., 42% rahb.	
90	1	<1%	Metavivianite	Bladed flaky crystals	1	1	<1% metaviv., <1% rhab.	Metaviv: bladed flaky crystals, rhab.: flakes & needle-like
	2	<1%	Metavivianite		2	1	<1% metaviv., <1% rhab.	
	5	<1%	Metavivianite		5	9	5% metaviv., 4% rhab.	
	28	100	64% rhab., 32% giniite, 4% monz.	Rhab: flakes & nano spherulites gin.: polygonal prisms, monz.: polygonal composite prisms	28	100	5% metaviv., 57% rhab., 38% giniite	Metaviv: bladed flaky crystals, rhab.: flakes, needle-like & nano-spherulites
165	0.2	99	94% giniite, 4% rhab.,2% hema.	Gin.: polygonal prisms; rhab. flakes & nano spherulites hema. nm polygonal prism aggregates	0.2	100	81% giniite, 16% rhab., 3% hema.	Gin.: polygonal prisms; rhab: flakes & nano spherulites, hema.: nm polygonal prism aggregates
	2	100	85% giniite, 10% rhab., 3% hema., 2% monz.	Gin.: polygonal prisms flakes & nano spherulites hema.: nm polygonal prism aggregates monz.: polygonal composite prisms	2	100	74% giniite, 5% rhab., 7% hema., 13% monz.	Gin.: polygonal prisms flakes & nano spherulites, hema.: nm polygonal prism aggregates, monaz.: polygonal composite prisms
	7	100	50% giniite, 11% rhab., 19% hema., 20% monz.	Gin.: polygonal prisms flakes & nano spherulites hema.: nm polygonal prism aggregates monz.: polygonal composite prisms	7	100	40% giniite, 8% rhab., 19% hema., 33% monz.	Gin.: polygonal prisms flakes & nano spherulites hema.: nm polygonal prism aggregates monz.: polygonal composite prisms

a Metavivianite (metaviv.), rhabdophane (rhab.), giniite (gin.), monazite (monz.), hematite (hema.).

The extent of the replacement reaction was found to be time and temperature dependent. At 50 and 90 °C we observed the gradual transformation of vivianite to metavivianite and the subsequent precipitation of rhabdophane (Table 1). At 90 °C the formation of giniite was recorded after 28 days. At 165 °C, the vivianite to metavivianite transformation was not observed due to the extremely fast transformation rate, and all of the host vivianite recrystallizing in less than 4 hours.

The observed mineralogy was also found to be temperature-and REE ratio-dependent. At 50 °C, transformation of vivianite to metaviviante proceeded at a faster rate in the equal concentration solutions compared to the PAAS solution. For example, 28% of the vivianite was consumed after one day in the equal concentration compared to 12% in the PAAS solution. At the end of the experiment (32 days), 60% of the vivianite had been consumed in the equal concentration solution compared to 45% in the PAAS solution.

At 90 °C, transformation of vivianite to metavivianite was slower compared to 50 °C, with less than 1% of vivianite consumed in the first 2 days. However, in contrast to the 50 °C experiments, it was observed that the PAAS solution experiments progressed at a slightly increased rate with 9% of vivianite consumed after 5 days compared to less than 1% in the equal concentration solution. There were also minor amounts of rhabdophane (1–4%) precipitation in the early stages (1–5 days) of the PAAS solution experiments compared to no rhabdophane detected in the equal concentration experiments after the same time.

At 165 °C, the initial solution concentrations played a less critical role. Similar consumption rates (<4 hours) of vivianite were recorded in both equal concentration and PAAS solutions. The subsequent transformation of vivianite to metavivianite, giniite and hematite as well as the precipitation of rhabdophane and its transformation to monazite occurred almost in parallel when comparing the equal concentration and PAAS solution experiments.

### Scanning electron microscopy – energy dispersive spectroscopy (SEM-EDS)

**Fig. 2:**
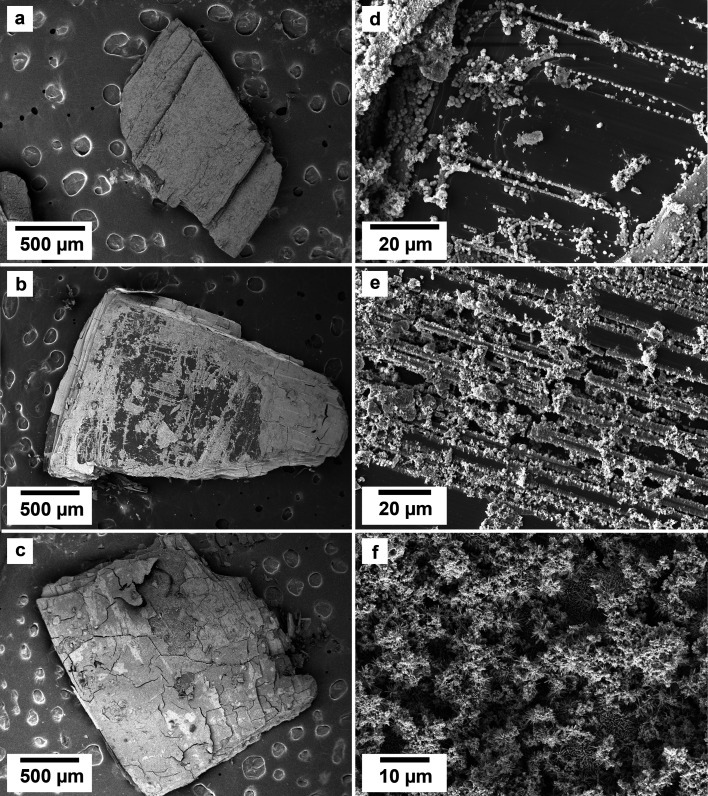
SEM micrographs of the surface of vivianite grains exposed to equal concentration REE-bearing solutions at 50 °C showing (a–c) the surface appearance due to the replacement of the vivianite by newly formed crystals; images (d–f) show details of the micro- and nano-crystalline materials gradually covering the host grain.

**Fig. 3:**
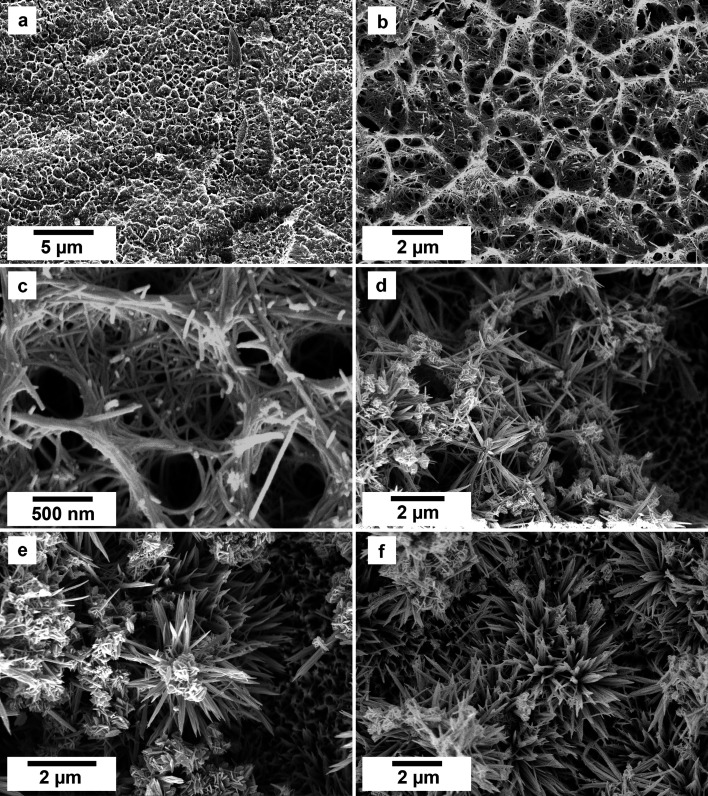
SEM photomicrographs showing the nanophases obtained in the experiments using equal concentration of REEs at 50 °C. (a–c) Surface of vivianite covered by metavivianite nanoaggregates; (d–f) growth of rhabdophane needle aggregates on the surface of the metavivianite.

**Fig. 4:**
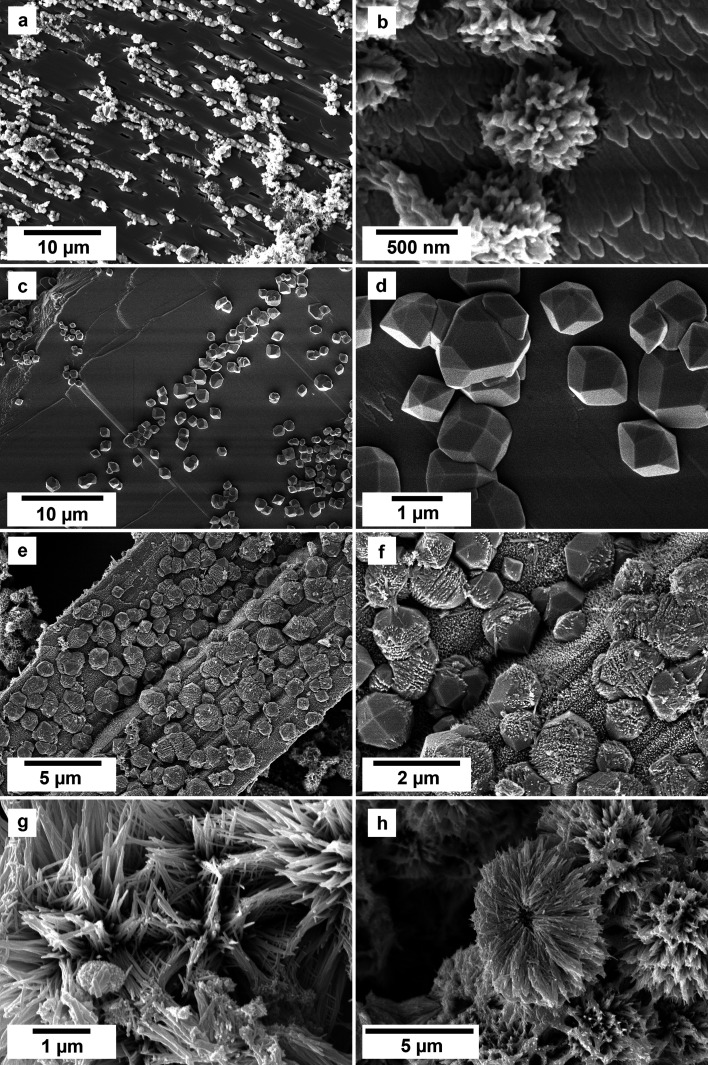
SEM photomicrographs showing the nanocrystalline solids obtained in the experiments at 90 °C using equal REE concentration solutions. (a and b) Surface of vivianite becoming covered by metavivianite nanoaggregates; (c and d) growth of giniite crystals on the surface of the vivianite; (e and f) early stages of growth of rhabdophane on the surface of giniite. (g and h) Rhabdophane nanoaggregates and spherulites covering the surface of the host crystals.

**Fig. 5:**
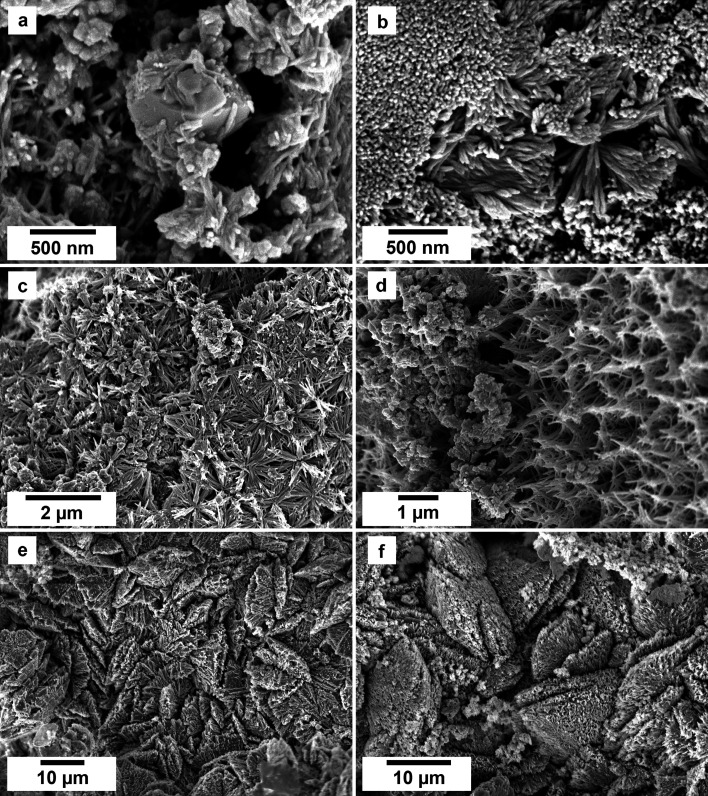
SEM photomicrographs showing the nanocrystals obtained in the experiments at 165 °C in the PAAS solution experiments. (a) Surface of giniite becoming covered by rhabdophane nanorods; (b and c) growth of rhabdophane nanocrystals on the surface of the host, sometimes forming spherulitic-type aggregates (c); (d) nanocrystals of hematite on rhabdophane. (e and f) Monazite and rhabdophane aggregates.

SEM imaging revealed the formation of crystalline phases on the surface of the vivianite grains. These newly formed phases were observed as surface precipitates that partially or fully covered the vivianite host grains. In some experiments, vivianite was fully replaced from the periphery inwards (Fig. 2). SEM photomicrographs showed that metavivianite initially formed in the dissolution pits of vivianite (Fig. 4a and b) and consisted of acicular aggregates with individual needles not exceeding lengths larger than 1 μm and thicknesses of ∼50 nm. At 50 °C the crystals looked acicular (Fig. 3a–c), at higher temperatures their morphology during the early stages of crystallization consisted mainly of clusters of irregular flakes with random orientation. At 50 °C we also observed the growth of nanometric flakes and acicular crystals of rhabdophane on the surface of metavivianite (Fig. 3d–f). The rhabdophane crystals were needle-like and sometimes formed a radiating pattern around a central point. However, these showed a less than perfect symmetrical orientation resulting in a slightly irregular, but still overall radiating appearance (Fig. 3e and f). Each individual crystal from the radiating structure had a length < 1 μm and a thickness of no more than 100 nm. At 90 °C we observed the growth of giniite crystals along with metavivianite on the surface of vivianite (Fig. 4c and d). The giniite crystals consisted of individual equant polygonal prisms with sizes between 600 nm and 2 μm. At 165 °C nanorods of rhabdophane began to cover the surface of giniite (Fig. 5a), sometimes forming spherulitic-type structures (Fig. 5b and c). Nanocrystals of hematite were also observed on the surface of rhabdophane. These consisted of nm polygonal prism aggregates with sizes < 200 nm (Fig. 5d). Finally, monazite presented as elongated composite prisms with polygonal consisting of acicular or flaky subunits. These subunits were aligned parallel or subparallel to the prism's elongation, giving the overall crystal a textured or layered appearance. The subunits had lengths between 500 nm and 2 μm and created a fibrous or platy surface on the prism's faces, reflecting their acicular or flaky nature (Fig. 5e and f).

SEM-EDS maps showed relatively homogenous uptake of the five REEs in the equal concentration solution experiments (ESI – Image 1†). In contrast, in the PAAS solution experiments the uptake of REE reflect their original concentration in the aqueous solution, with higher concentrations of La, Ce, and Nd (ESI – Image 2†).

## Discussion

The dissolution of vivianite and the subsequent formation of iron phosphates metavivianite and giniite, iron oxide hematite and REE-phosphates rhabdophane and monazite were the result of three simultaneous processes: (i) the solution-mediated oxidation of vivianite during its dissolution, coupled with the formation of secondary iron phosphates, (ii) the replacement of the iron phosphates by REE phosphates, and (iii) REE phosphate mineral transformation reactions. In fact, all three processes were mediated by the aqueous solution, with pH, temperature, and the ionic radius of the rare earths present in solution, playing key roles in the extent and rate of the replacement reactions.

### The progressive and concurrent oxidation and dissolution of vivianite

Under oxic conditions, vivianite is unstable, and structural Fe^2+^ oxidizes rapidly to Fe^3+^. While the vivianite crystal structure seems to maintain stability until an oxidation degree of ∼50%,^22^ the additional positive charge of the Fe^3+^ needs to be balanced by the conversion of a H_2_O ligand into an OH^−^ group and the release of a H^+^. Therefore, upon further oxidation of Fe^2+^ above the 50% threshold, the crystal structure is impacted, leading to significant surface modification, structural distortion, and phase changes.^23–25^ In our experiments, the progressive oxidation of vivianite resulted in the formation of metavivianite and giniite, where 66% and 80% of the Fe is Fe^3+^ respectively, and hematite (100% Fe^3+^). Crystallisation of these phases was the result of the solution-mediated coupled oxidation and dissolution of vivianite. This is a complex process that involves the interplay between oxidation reactions and the solubility of vivianite in aqueous environments and is strongly influenced by the aqueous solution chemistry, pH, and temperature.

The oxidation of vivianite crystals occurs more rapidly in solution compared to exposure to air due to enhanced availability of reactants, favourable environmental conditions, and different kinetic mechanisms that facilitate faster oxidation rates. In aqueous environments, the presence of dissolved oxygen (DO) significantly enhances the oxidation kinetics of Fe^2+^ ions in vivianite. Several studies indicate that the oxidation of Fe^2+^ to Fe^3+^ is also facilitated in solution due to the higher concentration of reactive species, which can lead to faster reaction rates compared to the limited diffusion of oxygen in air.^26,27^ Additionally, the kinetics of oxidation in solution are governed by different mechanisms than those occurring in air. In air, the oxidation process is primarily limited by the diffusion of oxygen in the mineral structure, which can create a slower reaction environment. In contrast, in solution, the oxidation can occur through direct contact with dissolved oxygen, leading to a more rapid dissolution and transformation of vivianite.^28,29^ Furthermore, the layered structure of vivianite, which is stabilized by hydrogen bonds, can be disrupted more easily in solution, allowing for easier access of oxidizing agents to the Fe^2+^ sites.^29,30^ The interaction of vivianite with water creates a dynamic environment where the solubility of oxygen is higher, allowing for more efficient oxidation processes. The aqueous medium also allows for better thermal management, as temperature fluctuations can be more easily controlled, thereby influencing the rate of oxidation reactions.^14^

The kinetics of oxidation were found to be temperature-dependent with metavivianite more abundant at 50 °C. At 90 °C, small amounts of metavivianite were recorded, however, after 28 days all metavivianite had transformed to giniite, a phase that was not seen at 50 °C. At the highest temperature of 165 °C, no metavivianite was detected, giniite was the main phase with around 20% transformed to hematite. Increased temperature significantly affects the oxidation of vivianite in aqueous environments by accelerating the reaction kinetics and altering the mineral's stability. As temperature rises, the solubility of gases, including oxygen, decreases, but the diffusion rate of oxygen in water increases, which enhances the oxidation rate. This can lead to enhanced oxidation rates of Fe^2+^ ions present in vivianite. At elevated temperatures (155–185 °C), such as those observed by Wang *et al.*, vivianite will undergo partial transformation into various iron phosphate phases such as strengite and giniite. This auto-oxidation is facilitated by the decomposition of water at higher temperatures.^31^ The increased thermal energy not only enhances the mobility of ions but also promotes the breaking of chemical bonds within the vivianite structure, thereby accelerating the oxidation process.^32^ Studies have shown that even moderate increases in temperature can compromise the structural integrity of vivianite leading to significant alterations in the crystal structure, subsequently enhancing its susceptibility to oxidation.^14^ For example, the presence of bound water in the vivianite crystal plays a crucial role in stabilizing the structure, however, as temperature rises, the hydration layer can be disrupted, making the Fe^2+^ ions more accessible to oxidizing agents.^33^ In our experiments, vivianite partially dissolved at 50 °C, however at higher temperatures, vivianite was fully replaced by giniite and rhabdophane (90 °C) and giniite, hematite, rhabdophane, and monazite (165 °C). This is in agreement with Metz, *et al.*, 2024 who experimentally demonstrated that the maximum temperature at which vivianite is stable under oxic conditions is between 65 and 75 °C.

The slower transformation kinetics at 50 °C could also be explained by a partial equilibrium situation in which a crust of nano-sized metavivianite covering the vivianite host hinders further oxidation and dissolution.^34–36^ Similar results were recorded by Yu *et al.*, 2023 in their investigation of the effects of dissolved oxygen (DO) concentrations on As(v) adsorption onto the iron carbonate siderite. They found that excess DO caused fast oxidation of siderite which in turn constrained As(v) adsorption. This was due to the production of a coating of Fe(iii) oxides on the surface of the siderite that prevented any further oxidation of the host.^37,38^

The oxidation of vivianite in water is also significantly influenced by pH, which affects both the stability of the mineral as well as the kinetics of oxidation reactions through the alteration of iron and phosphate speciation. When vivianite is oxidised, the Fe^2+^ and phosphate ions present in its structure can be released into the aqueous phase, leading to the dissolution of vivianite. The oxidation of Fe^2+^ to Fe^3+^ results in the release of protons (H^+^) into the solution, which will initially lower the pH. At low pH, extensive protonation at the vivianite surface results in the weakening of structural Fe–(PO_4_) bonds due to polarization of water molecules promoting Fe detachment.^39^ The Fe can then undergo hydrolysis and lead to the formation of various hydrolysed ferrous species, such as Fe(OH)^+^ and Fe(OH)_2_^+^. This has been experimentally shown to be dependent on the pH of the solution. Morgan and Lahav described the fundamental equilibrium and thermodynamic principles governing the kinetics of ferrous iron (Fe^2+^) to ferric iron (Fe^3+^). They propose a pH dependent range of ∼5 < pH < ∼8 and a pH independent range of ∼5 > pH > ∼8. In other words, the overall oxidation rate depends on the distribution of these three species in the aqueous phase, which is pH dependent. At pH below ∼4, Fe^2+^ concentration dominates, and the rate is independent of pH. At pH above ∼5, [Fe(OH)_2_] determines the rate because it is far more readily oxidised than both Fe^2+^ and FeOH^+^. Between pH 5 and 8, the Fe(OH)_2_ concentration rises steeply with pH and the overall oxidation rate increases accordingly. Finally, at pH > ∼8 [Fe(OH)_2_] no longer varies with pH and the oxidation is again independent of pH.^40^ This is in agreement with Luther, 1995 who used molecular orbital theory to show that hydrolysed ferrous iron species are more readily oxidised than non-hydrolysed ferrous species:Fe(OH)_2(aq)_ ≫ Fe(OH)^+^ ≫ Fe^2+^

However, as the oxidation progresses and ferric iron precipitates, the overall effect can lead to an increase in pH due to the consumption of protons during the formation of insoluble iron oxides.^41^

### Integration of PHREEQC modelling with the experimental results

PHREEQC calculations provided valuable insights into the stability and transformation of Fe- and REE-bearing phases during the interaction of vivianite with REE aqueous solutions. The calculated saturation indices confirmed the thermodynamic favourability of iron oxyhydroxide precipitation, with Fe(OH)_3_, goethite, and hematite exhibiting high SI values across all conditions. Notably, the high SI of hematite (+24.82 at 50 °C) aligns with our experimental observations of progressive oxidation from vivianite to giniite and ultimately to hematite at higher temperatures. Similarly, REE-phosphates such as rhabdophane and monazite displayed variable SI values depending on the specific REE present, with Ce-monazite reaching an SI of 9.91 at 50 °C, suggesting preferential formation under equilibrium conditions.

The PHREEQC-derived pH trends further support our experimental findings regarding phase stability and transformation. The initial solution pH (∼5.1) increased to ∼6.93 at 50 °C, promoting Fe hydrolysis and the subsequent oxidation of Fe(OH)_2_. At this temperature, vivianite remained relatively stable, preventing its complete dissolution and full transformation into metavivianite and rhabdophane. As the temperature increased to 90 °C, the pH remained stable (∼6.90), but here, the conditions exceeded the previously reported vivianite stability zone (65–75 °C). Consequently, after 28 days, all vivianite had fully oxidised and dissolved, leading to the crystallisation of giniite and rhabdophane. At 165 °C, the pH slightly decreased (∼6.50) but remained within the favourable range for Fe hydrolysis and oxidation. Under these conditions, vivianite dissolution was rapid and complete, with any unincorporated Fe^3+^ precipitating as hematite. This transition from vivianite to giniite and hematite, along with the precipitation of REE-phosphates, is well supported by both experimental observations and PHREEQC predictions.

The stability of rhabdophane under all temperature conditions aligns well with the PHREEQC-calculated saturation indices, reinforcing its role as a key intermediate phase in REE-phosphate formation. However, at 165 °C, a transformation from rhabdophane to monazite was experimentally observed. This transformation is driven by thermal dehydration, structural rearrangement, and increasing thermodynamic stability of monazite at elevated temperatures. While rhabdophane is more soluble at lower temperatures, monazite exhibits significantly lower solubility and greater density, making it the thermodynamically favoured phase at 165 °C. This transformation is also influenced by the ionic radius of REEs, where larger REEs such as La, Ce, and Nd promote earlier dehydration and structural collapse of rhabdophane.

Despite cerianite (CeO_2_) consistently exhibiting positive SI values in PHREEQC calculations, its formation was not observed in our experiments. This is because the oxidation of Ce^3+^ to Ce^4+^ is not strongly favoured at near-neutral pH compared to alkaline or highly basic conditions.^42–44^ Under the experimental conditions used in this study, Ce remained in the trivalent state, leading to the preferential precipitation of Ce-bearing monazite rather than cerianite. If the pH were to evolve into the basic range, the oxidation of Ce^3+^ to Ce^4+^ would become thermodynamically favourable, promoting the dissolution of Ce-bearing phosphates. Under such conditions, Ce^4+^ could lead to the destabilisation and subsequent dissolution of rhabdophane and monazite in contact with the solution. This behaviour is analogous to the destabilisation of Ce-bearing carbonates under alkaline conditions.^42–45^

The strong agreement between PHREEQC modelling and experimental results validates the oxidation-driven pathway from vivianite to giniite and hematite while confirming the stability and transformation mechanisms of REE-phosphates under varying conditions.

### Replacement of iron phosphates by rare earth element phosphates

The replacement of iron phosphates by rhabdophane, a hydrated rare earth phosphate, can occur through several mechanisms, including dissolution–precipitation reactions. When iron phosphates, like vivianite, metavivianite and giniite dissolve in the presence of rare earth-bearing solutions, the released phosphate ions can react with dissolved REE ions to form rhabdophane. The presence of REEs has been shown to stabilize the formation of rhabdophane through ionic interactions. The effect of the REE^3+^ ionic radius on the formation and stability of rhabdophane is a significant factor in understanding its crystallization behaviour and thermodynamic properties. One key observation is that the ionic radius of the rare earth ion directly affects the stability of rhabdophane. Larger lanthanide ions, such as La^3+^–Gd^3+^ with ionic radii ranging from 1.250–1.105 Å,^46^ are more favourable for the formation of rhabdophane due to their ability to stabilize the crystal lattice.^47^ Smaller lanthanide ions, such as Gd^3+^–Lu^3+^ with ionic radii ranging from 1.105–0.995 Å,^46^ tend to destabilize the rhabdophane structure. The incorporation of smaller ions can lead to an increased lattice strain and reduced stability, making the rhabdophane more susceptible to transformation into other phosphate structures, such as monazite or xenotime.^48^

With time, we recorded the replacement of the iron phosphates, metavivianite and giniite, with REE-phosphates rhabdophane and monazite. As the vivianite dissolution progressed, releasing phosphate ions into the solution, it created conditions favourable to REE-phosphate precipitation. Normally these conditions would be governed by the solubility products of the initial vivianite and the subsequent newly formed rhabdophane and monazite. However, the solubility products for vivianite are lg *K*_sp_ = −35.76 ± 0.076 (ref. 49) and range for rhabdophane from lg *K*_sp_ = −25.6 ± 0.8 (Pr) to −24.9 ± 1.7 (Eu).^50^ As rhabdophane is more soluble than vivianite, it should not be forming in these conditions. We, therefore, propose that the crystallization processes happening in our experiments are driven by the oxidation of vivianite and secondary Fe^2+^-bearing phosphates. Equilibrium with respect to vivianite is never reached as the majority of Fe^2+^ in solution is constantly oxidising to Fe^3+^. The process is enhanced by the higher temperatures, which promotes further dissolution of the host, enriching the fluid with PO_4_ and more Fe^2+^, which, again, will oxidise to Fe^3+^. As the oxidation process is progressive and not instantaneous, some of the Fe^2+^ will incorporate in the metastable phases metavivianite and giniite. As the system progresses, the fluid becomes highly enriched in PO_4_ because vivianite is unable to reach equilibrium with the aqueous solution, increasing the supersaturation levels for the REE phosphates. The PO_4_ will therefore combine with the REE in solution to form rhabdophane which at some point will transform to monazite as temperatures increased from 90 to 165 °C, contributing to the depletion of phosphate in the aqueous solution and the crystallisation of iron oxides. The observed behaviour is in agreement with Gausse *et al.*, 2016, who noted that the solubility of rhabdophanes is influenced by thermodynamic equilibria, which can shift in favour of rhabdophane precipitation when iron phosphates are dissolved. They demonstrated that in aqueous environments at temperatures below 90 °C, rhabdophane is more stable than the corresponding monazite-type phase.

Supersaturation levels at the host-mineral–solution interface would also contribute to the rapid crystal nucleation of secondary REE-phosphates. The dissolution of only a few monolayers of the surface host is sufficient enough to initiate the formation of a fluid boundary layer near the surface of the host seed, resulting in the development of a surface precipitate.^51^ The high concentration of PO_4_ ions at the fluid boundary layer of the vivianite, metavivianite and giniite compared to the bulk solution promoted the high supersaturation levels required for the spherulitic crystals of rhabdophane that we observed in our 165 °C experiments.

It has also been shown that the presence of iron oxyhydroxides play a role in the adsorption and desorption processes that govern the availability of REE and phosphates in solution. Beyond the formation of specific iron and REE phosphate minerals, the surface properties of the iron-bearing phases formed during vivianite oxidation also play a crucial role in phosphate and REE mobility and availability. Fe^2+^ minerals, like vivianite, generally exhibit a lower surface charge density and a different surface affinity for phosphate compared to Fe^3+^ minerals, such as hematite or the iron oxyhydroxides that form during vivianite oxidation. As vivianite (Fe^2+^) oxidizes and transforms into metavivianite, giniite (mixed Fe^2+^/Fe^3+^), and ultimately hematite (Fe^3+^) and iron oxyhydroxides, the dominant surface chemistry shifts, promoting the formation of rhabdophane and monazite, therefore contributing to depletion of phosphate in the aqueous solution and the crystallization of iron oxides. Belogub *et al.*, 2021 discussed how the interaction between iron oxyhydroxides and phosphate ions can influence the formation of rhabdophane, as these interactions can affect the mobility and concentration of REE in the environment. It is the sorption of REE on iron oxyhydroxides that cause early REE scavenging from solutions, with a pH > 5.^52^ Sorption and desorption of REEs on iron oxyhydroxides depend on particle size and morphology, crystallographic defects, composition, and purity of the sorbent surface.^53^ Concomitantly, adsorption capacity also strongly depends on the pH and the ionic strength of the solution. The combination of these properties determines whether adsorption takes place *via* the formation of outer-sphere or inner-sphere complexes.^54,55^ REE adsorption on iron oxyhydroxides, occurring before rhabdophane formation, can involve both inner-sphere and outer-sphere complexation mechanisms. The specific type of complexation plays a key role in determining the thermodynamic stability and crystal properties of rhabdophane. Inner-sphere complexes are characterized by direct coordination between the rare earth ions and the surface hydroxyl groups of iron oxyhydroxides. This type of complexation typically results in stronger chemical bonds, leading to enhanced stability of the adsorbed species. Studies have shown that the inner-sphere complexes are less sensitive to changes in ionic strength of the aqueous solution, making them more stable under varying environmental conditions.^56^ For example,^57^ indicated that inner-sphere adsorption sites are strong and stable, which is crucial for the retention of REEs in solid phases such as rhabdophane. The formation of inner-sphere complexes on iron oxyhydroxides can also contribute to the overall stability of rhabdophane by reducing the mobility of REEs, thereby limiting their release back into solution.^58^ In contrast, outer-sphere complexes involve weaker electrostatic interactions where REE ions are held at the surface of the iron oxyhydroxides *via* water molecules without forming strong chemical bonds. This type of complexation is more sensitive to ionic strength. As ionic strength increases, the stability of the outer-sphere complexes can decrease due to competition with other ions in solution.^59^ The lower stability of outer-sphere complexes can lead to higher desorption rates and reduced retention of REEs in the environment.^56^ The presence of both types of complexes in the formation of rhabdophane suggests that the stability of the mineral can be influenced by the conditions in which it forms. For example, at lower pH values (∼4–7), the competition between REEs and protons can lead to increased outer-sphere complexation, while higher pH levels (∼7–9) promote inner-sphere complexation due to the availability of hydroxyl groups for coordination.^60^ For each of our temperature parameters, 50, 90, and 165 °C, the pH of the aqueous solution evolved from an initial value of 5.1 (before vivianite was added to the aqueous solution) to ∼6.93, 6.90, and 6.50 respectively. These pH values would allow for the more stable inner-sphere complexation, and this would be consistent with the formation of rhabdophane at all temperatures. Also, the slightly lower value of the pH 6.50 at 165 °C (compared to 6.90 at 90 °C) could also promote for the initiation of rhabdophane transformation to monazite.^61^

### Transformation of rhabdophane to monazite

Rhabdophane, a hydrated rare earth phosphate, can transform to monazite, an anhydrous rare earth phosphate. The transformation of rhabdophane to monazite is influenced by several factors, including temperature, pH, REE speciation and ionic radii, hydration state, and differences in solubility (Pan *et al.*, 2024). At higher temperatures hydrous rhabdophane will undergo dehydration, and the structural changes will facilitate its conversion to the more thermodynamically stable anhydrous monazite.^62^ Full dehydration towards anhydrous rhabdophane takes place between 190 and 240 °C, with the irreversible transformation into monazite in aqueous solution occurring at temperatures above 500 °C.^63^ The rhabdophane crystal structure presents as hexagonal symmetry and consists of alternating PO_4_ tetrahedra and LnO_8_ polyhedral linked together in chains along the [100] direction creating large one-dimensional channels, which accommodate the water molecules. Monazite presents as monoclinic symmetry with a crystal structure comprised of PO_4_ tetrahedra and LnO_9_ polyhedral also linked together in chains along the *c*-axis, but in a much more compact way.^64^ The transformation process involves the progressive loss of water from larger channels along the *c*-axis of the rhabdophane structure, leading to the conversion to monazite.^65,66^ This loss of water molecules is crucial for stabilizing the monazite structure and can commence within the temperature range of 110–400 °C. This leads to the formation of anhydrous hexagonal CePO_4_, before transitioning to the monoclinic form of CePO_4_, monazite.^67^ The thermal stability of monazite at high temperatures is well documented, with full transformation from rhabdophane completed at temperatures > 900 °C when heated in air.^68^ However, it has been reported that the process can be initiated at significantly lower temperatures, particularly in the presence of water^68^ and can also occur in samples that have been heat-treated during synthesis.^65^ While solubility values vary in the literature for monazite at our experimental conditions, all are in agreement that monazite is less soluble than rhabdophane at temperatures of 165 °C.^48,69–71^ Results from Van Hoozan *et al.*, also demonstrate that the solubility of monazite end members, LaPO_4_, PrPO_4,_ NdPO_4_, and EuPO_4_, decrease sharply with increasing temperatures between 100 and 250 °C.

The transformation kinetics from rhabdophane to monazite can also be influenced by the ionic radius of the rare earth elements involved. Shelyug *et al.* demonstrated that the transformation temperature decreases with an increase in the ionic radius of the lanthanides. For example, when heated in air, the temperature required to transform the larger La-bearing rhabdophane to monazite is ∼750 °C, compared to 900 °C to fully transform the smaller Gd-bearing rhabdophane.^48^ This is in agreement with^72^ who demonstrated that the smaller, heavier REEs tend to form inner-sphere complexes. The formation of inner-sphere complexes typically results in a more stable adsorption site, requiring higher heat for transformation.

In our experiments at 165 °C, we recorded the initiation of the transformation of rhabdophane to monazite. However, as the temperature is well below the reported temperature range of 700–900 °C for complete transformation in air and 500 °C in aqueous solution, we observed only a 20 and 30% transformation in the equal concentration and PAAS solutions respectively. We propose that while the transformation was initiated by the dehydration of rhabdophane, it is the solution chemistry containing mainly larger REE ions La, Ce, Pr, Nd, with ionic radii of 1.250, 1,220, 1,200, and 1.175 Å respectively, that helped to facilitate the transformation at relatively lower temperatures. As stated above, larger, lighter REEs tend to form outer-sphere complexes, and can begin to transform at lower than expected temperatures. SEM-EDS maps showed a relatively homogenous uptake of the REEs in the equal concentration experiments. However, in the PAAS solution experiments there was a preferential uptake of the larger, lighter REE which also correlated to their original concentration in the aqueous solution, with higher concentrations of La, Ce, and Nd. In contrast, point analysis revealed no Dy incorporation into the rhabdophane. This could be the result of its low concentration in the PAAS solution as well as its incompatible smaller ionic radii of 1.075 Å^46^ (ESI Images 1 and 2†).

## Conclusion

The interaction of vivianite grains with multi-component REE-aqueous solution resulted in the progressive oxidation and dissolution of vivianite and its transformation to iron phosphates, metavivianite and giniite and iron oxide hematite. The rate of vivianite dissolution increased with increasing temperature, with metavivianite and giniite being replaced by the rare earth phosphate rhabdophane. The pH range of ∼6.5–6.93 maintained the stability of rhabdophane for all three experimental temperatures (50, 90, and 165 °C). At the highest temperature of 165 °C we observed the partial transformation of rhabdophane to monazite. The transformation initiated below the documented temperature of 500 °C for complete transformation. Therefore, we propose that it is the incorporation of larger REE and their tendency to form more weakly bound outer-sphere complexes. The transformation of iron phosphate, such as vivianite, into more stable and less soluble REE-phosphates has potential applications in REE recovery, particularly in waste materials, contaminated soils, and mine waste waters.

## Data availability

The data that support the findings in this study are available from the corresponding author upon reasonable request.

## Conflicts of interest

The authors declare no conflict of interest.

## Supplementary Material

RA-015-D4RA08110B-s001
