# Supplementary material for: Nanophase REE phosphate crystallization induced by vivianite oxidation: mechanistic insights and mineralogical implications
Source: RSC Adv. 2025 Apr 9;15(14):11257–70. doi: 10.1039/d4ra08110b (PMC11979900; doi:10.1039/d4ra08110b)
Supplement: RA-015-D4RA08110B-s001 [file RA-015-D4RA08110B-s001.pdf]

Supplementary Information (SI) for RSC Advances.
This journal is © The Royal Society of Chemistry 2025

Supporting information for the paper

Nanophase REE Phosphate Crystallization Induced by Vivianite Oxidation: Mechanistic Insights and Mineralogical Implications

Maddin, M.,*[a] Terribili, L.,[a] Rateau, R., Szucs, A.M.,[a] and Rodriguez-Blanco, J.D[b]

[a]*Department of Geology, School of Natural Sciences, Trinity College Dublin, Dublin 2, Ireland*

[b]*iCRAG, Department of Geology, School of Natural Sciences, Trinity College Dublin, Dublin 2, Ireland*

* *email: maddinm@tcd.ie*

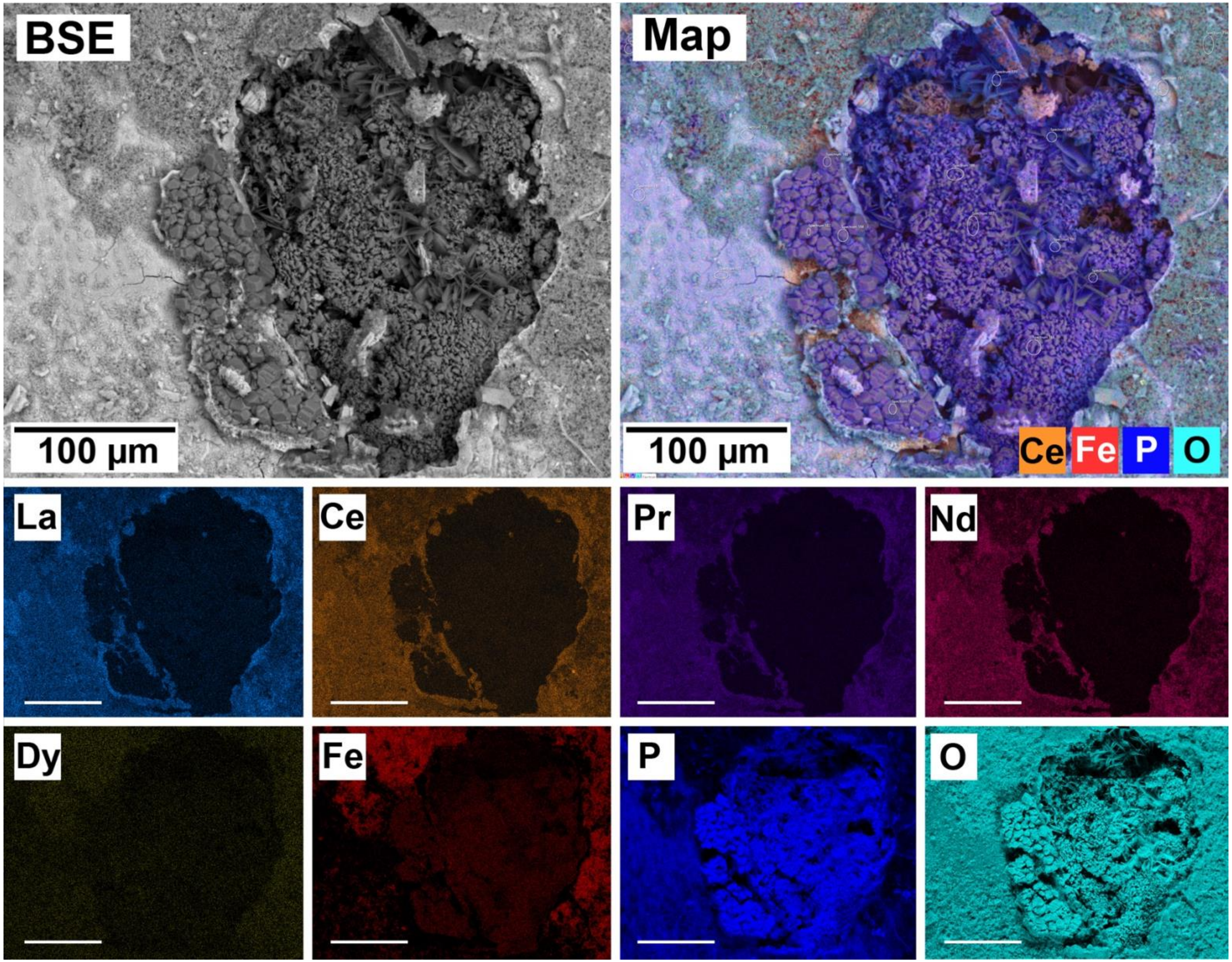

Figure SI-1: EDS elemental maps of sample obtained after 148 hours in the equal concentrations experiment at 165 ºC.

Table SI-1: EDS elemental analyses of sample obtained after 148 hours in the equal concentrations experiment at 165 ºC.

Equal Concentration	C	O	P	Fe	La	Ce	Pr	Nd	Dy	Total
Spectrum 175	26.36	52.73	4.43	11.27	0.8	1.02	1.26	1.2	0.94	100
Spectrum 176	25.2	50.39	1.99	20.53	0.24	0.4	0.4	0.47	0.4	100
Spectrum 177	24.86	49.71	6.93	16.6	0.32	0.62	0.56	0.4	0	100
Spectrum 178	24.1	48.2	1.18	24.63	0.21	0.46	0.47	0.28	0.46	100
Spectrum 179	25.79	51.59	7.43	7.44	0.98	1.75	1.96	1.58	1.49	100
Spectrum 180	24.75	49.51	9.31	11.3	0.54	1	1.3	1.16	1.12	100
Spectrum 181	24.88	49.75	8.6	11.24	0.71	1.23	1.32	1.19	1.09	100
Spectrum 182	27.25	54.49	7.91	10.35	0	0	0	0	0	100
Spectrum 183	26.45	52.91	7.94	12.7	0	0	0	0	0	100
Spectrum 184	26.69	53.38	7.95	11.99	0	0	0	0	0	100
Spectrum 185	26.82	53.63	7.96	11.59	0	0	0	0	0	100
Spectrum 186	25.82	51.65	8.28	14.25	0	0	0	0	0	100
Spectrum 187	25.72	51.44	8.24	14.6	0	0	0	0	0	100
Spectrum 188	25.61	51.22	8.49	14.68	0	0	0	0	0	100
Spectrum 189	24.7	49.39	7	18.91	0	0	0	0	0	100
Spectrum 190	25.99	51.97	7.23	14.81	0	0	0	0	0	100
Spectrum 191	25.35	50.69	8.28	15.67	0	0	0	0	0	100
Spectrum 192	26.36	52.72	7.44	13.49	0	0	0	0	0	100
Spectrum 193	23	46	6.48	14.63	1.34	2.04	2.45	2.2	1.86	100
Spectrum 194	24.71	49.41	1.71	21.41	0.41	0.61	0.64	0.56	0.54	100
Spectrum 195	24.5	49.01	1.69	22.78	0.25	0.48	0.48	0.38	0.42	100

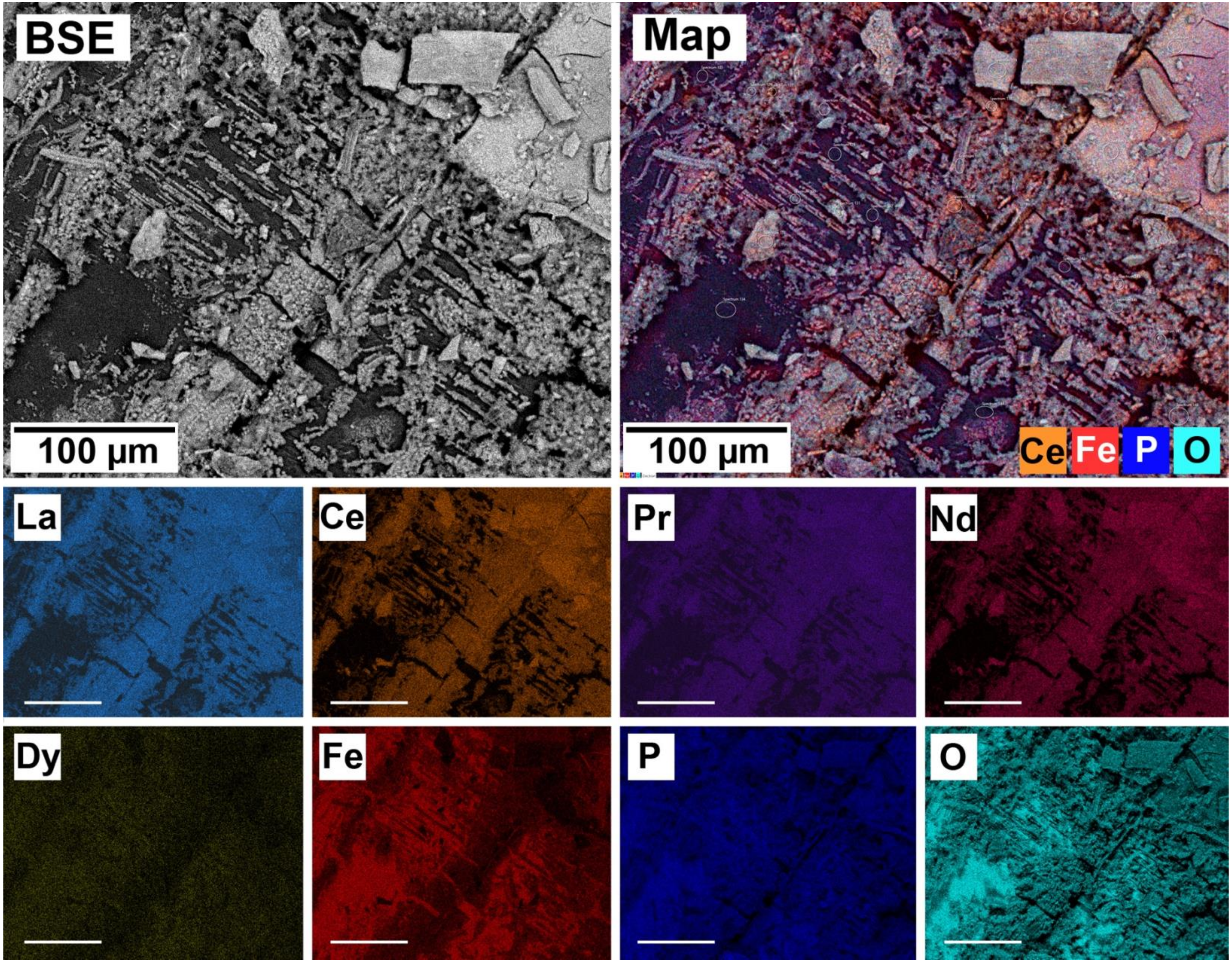

Figure SI-2: EDS elemental maps of sample obtained after 48 hours in the PAAS experiment at 90 ºC.

Table SI-2: EDS elemental composition of sample obtained after 48 hours in the PAAS experiment at 90 ºC.

PAAS 90 °C 48 hrs	C	O	P	Fe	La	Ce	Pr	Nd	Dy	Total
Spectrum 123	26.88	53.76	9.31	2.12	1.76	4.49	0	1.68	0	100
Spectrum 124	28.8	57.6	5.37	8.23	0	0	0	0	0	100
Spectrum 125	27.46	54.92	6.41	11.2	0	0	0	0	0	100
Spectrum 126	27.59	55.17	7.22	5.79	1.11	2.15	0	0.97	0	100
Spectrum 127	18.9	37.8	11.19	17.78	3.98	7.48	0	2.86	0	100
Spectrum 128	27.37	54.74	7.01	7.61	0.88	1.58	0	0.82	0	100
Spectrum 129	28.59	57.18	5.56	8.67	0	0	0	0	0	100
Spectrum 130	27.66	55.33	6.94	7.56	0.7	1.25	0	0.55	0	100
Spectrum 131	27.89	55.78	6.99	3.2	1.5	3.22	0	1.41	0	100
Spectrum 132	28.51	57.02	5.62	8.86	0	0	0	0	0	100
Spectrum 133	26.55	53.11	8.34	5.17	1.42	4.01	0	1.4	0	100
Spectrum 134	28.81	57.62	5.34	8.23	0	0	0	0	0	100
Spectrum 135	26.55	53.09	8.03	5.92	1.73	3.4	0	1.28	0	100
Spectrum 136	20.37	40.74	11.05	14.1	3.29	6.99	1.15	2.3	0	100
Spectrum 137	27.33	54.66	7.44	6.08	1.03	2.44	0	1.02	0	100
Spectrum 138	24.67	49.34	7.92	9.97	2.17	4.27	0	1.65	0	100
Spectrum 139	27.05	54.09	8.3	3.93	1.21	3.57	0.38	1.46	0	100
Spectrum 140	22.69	45.38	10.03	11.5	2.62	5.74	0	2.03	0	100
Spectrum 141	26.18	52.36	9.18	4.7	1.15	4.21	0.56	1.67	0	100
Spectrum 142	26.28	52.57	9.65	3.34	1.44	4.51	0	1.79	0.41	100
Spectrum 143	26.85	53.7	6.58	12.87	0	0	0	0	0	100
Spectrum 144	28.88	57.76	5.4	7.96	0	0	0	0	0	100
Spectrum 145	25.17	50.35	9.3	7.75	2.12	3.82	0	1.49	0	100
Spectrum 146	27.77	55.55	6.61	5.08	1.15	2.45	0.46	0.92	0	100
Spectrum 147	26.81	53.62	7.73	6.87	1.33	2.57	0	1.07	0	100
Spectrum 148	26.67	53.34	7.61	7.16	1.39	2.69	0	1.15	0	100
Spectrum 149	24.72	49.44	8.51	8.38	2.53	4.8	0	1.62	0	100
